# Chewing the Fat with Microbes: Lipid Crosstalk in the Gut

**DOI:** 10.3390/nu14030573

**Published:** 2022-01-28

**Authors:** Johanna M. S. Lemons, LinShu Liu

**Affiliations:** Dairy and Functional Foods Research Unit, Eastern Regional Research Center, Agricultural Research Service, United States Department of Agriculture, Wyndmoor, PA 19038, USA; linshu.liu@usda.gov

**Keywords:** gut microbiome, lipid, metabolism, intestinal health, mitochondria, nutrition, circadian rhythms

## Abstract

It is becoming increasingly important for any project aimed at understanding the effects of diet on human health, to also consider the combined effect of the trillions of microbes within the gut which modify and are modified by dietary nutrients. A healthy microbiome is diverse and contributes to host health, partly via the production and subsequent host absorption of secondary metabolites. Many of the beneficial bacteria in the gut rely on specific nutrients, such as dietary fiber, to survive and thrive. In the absence of those nutrients, the relative proportion of good commensal bacteria dwindles while communities of opportunistic, and potentially pathogenic, bacteria expand. Therefore, it is unsurprising that both diet and the gut microbiome have been associated with numerous human diseases. Inflammatory bowel diseases and colorectal cancer are associated with the presence of certain pathogenic bacteria and risk increases with consumption of a Western diet, which is typically high in fat, protein, and refined carbohydrates, but low in plant-based fibers. Indeed, despite increased screening and better care, colorectal cancer is still the 2nd leading cause of cancer death in the US and is the 3rd most diagnosed cancer among US men and women. Rates are rising worldwide as diets are becoming more westernized, alongside rising rates of metabolic diseases like obesity and diabetes. Understanding how a modern diet influences the microbiota and how subsequent microbial alterations effect human health will become essential in guiding personalized nutrition and healthcare in the future. Herein, we will summarize some of the latest advances in understanding of the three-way interaction between the human host, the gut microbiome, and the specific class of dietary nutrients, lipids.

## 1. Introduction

The quality and quantity of nutrients supplied to cells through food choices ultimately determines physical health. Metabolism links nutritional choices to cellular and body health through energy production, cell signaling, and epigenetics. It is generally appreciated that a fiber-rich diet is good for human health, with benefits such as increased intestinal motility, increased satiety, better glucose and cholesterol regulation, and lowered risk of intestinal diseases including colorectal cancer [1]. While fiber is indigestible by the human gut, there exists a diverse microbial community within the gastrointestinal tract that can utilize it and cross-feed the gut epithelium with secondary metabolites. Host–microbiota interactions along the gastrointestinal tract vary regionally; the swiftly moving, oxygenated luminal environment of the small intestine does not support as much microbial growth (10^3–8^ cells/mL) as the slower moving, oxygen depleted luminal environment of the colon (10^12^ cells/mL) [2]. Due to its proximity to these microbes and the physical and biochemical stresses of the digestive process, lots of cell turnover is required to maintain the integrity of the intestinal epithelium. The high energy demand and physical necessity of tissue turnover puts the intestinal epithelium at risk when dietary conditions do not support a healthy equilibrium. Unsurprisingly, dietary patterns of individuals, along with factors like antibiotic use, largely determine the composition of a gut microbial community [3]. While high levels of interindividual variation make it difficult to define a healthy microbiome, researchers agree that a dysbiotic microbiome puts the host at risk for multiple physical and emotional ailments, including Alzheimer’s disease, atherosclerosis, autism, depression, non-alcoholic fatty liver disease, inflammatory bowel diseases (IBD), and colorectal cancer [4,5,6,7,8,9]. It is hypothesized that some of the familial susceptibility to these diseases may result from a loss of microbial species diversity over generations which negatively affects host metabolism and epigenetics [10].

The typical Western diet is characterized by high consumption of dietary fats and refined carbohydrates and low consumption of plant-based fibers [11]. Human metabolism has adapted to handle short periods of undernutrition, which would have been advantageous throughout evolution. Humans are less adapted to overnutrition and many of the diseases associated with the Western diet (i.e., obesity, diabetes, cardiovascular disease) involve impaired metabolic switching between glucose and lipids or aberrant lipid storage [12]. These diseases are also associated with an altered gut microbiome leading to a causality dilemma. In trying to understand metabolism in the gut, how diet, the host, and the microbiota work together to influence health and disease must be considered [4,13]. In this review, we will pay special attention to recent advances in the understanding of lipid metabolism as it relates to the gut. We use the term “lipid metabolism” to broadly describe the bodily production, degradation, distribution, and storage of insoluble organic compounds like triglycerides (TGs), sterols, phospholipids, sphingolipids, fatty acids, etc. This review is meant to be a broad overview of the many ways in which the host and gut microbes interact with, utilize, and engage in crosstalk using lipids as a mediator.

## 2. Organization in the Intestinal Tract

The intestinal tract is composed of two distinct regions: the small intestine, whose main function is nutrient absorption, and the large intestine, whose main function is water absorption [14]. The intestinal epithelium, which will be described in more detail below, is physically supported by a mesenchymal layer consisting of intestinal fibroblasts and myofibroblasts, which also participate in cell–cell signaling that help regulate epithelial cell fate [15]. The gut is also home to many immune cells (B-cells, T-cells, macrophages, neutrophils) which have the difficult job of distinguishing friend from foe among the gut microbiota [16]. The gut microbiota is made up of organisms from all three domains of life—eukaryota, bacteria, and archaea—and its composition greatly impacts host homeostasis. Approximately 90% of the microbiota of healthy individuals is made up of bacteria from just two bacterial phyla, *Firmicutes* and *Bacteroidetes*; the remaining 10% is divided up among organisms from other phyla like *Actinobacteria*, *Proteobacteria*, *Fusobacteria*, and *Verrucomicrobia* [17]. These bacteria consume dietary nutrients and produce metabolites such as short chain fatty acids (SCFAs), B and K vitamins, secondary bile acids, and a variety of polyphenolic degradation products that impact the host. While the overall balance of these organisms in the gut is a common biomarker for health status, even low abundance bacteria can have a large impact [18,19].

The small intestinal lining is composed of a single layer of tightly associated epithelial cells that organize into invaginations into the lamina propria, known as crypts of Lieberkühn and villi that extend into the luminal space. At the base of each crypt are 5–7 active Lgr5+ intestinal stem cells (ISC), each surrounded by Paneth cells which supply growth signals and nutrients. The colon contains crypts, but no villi, and specialized enteroendocrine cells are thought to fulfill the same role as Paneth cells [14,20]. Long-term homeostasis of a tissue requires stem cells to balance self-renewal and differentiation. The high turnover rate of this tissue means ISC divide continuously, cycling through the cell cycle approximately every 21.5 h [21]. During differentiation, ISC produce lineage defined transit amplifying (TA) cells which divide several more times before fully differentiating into mature absorptive and secretory cells [22]. Those of the absorptive lineage differentiate into enterocytes (colonocytes in the large intestine) or M cells [16]. Enterocytes are the most abundant cell type in the intestinal epithelia, accounting for approximately 80% of the cells. Additionally, as we will cover later in this paper, there have been multiple reports of perturbations that shift the cellular balance toward the secretory lineage which produces differentiated goblet cells, Paneth cells, tuft cells and enteroendocrine cells [16,23]. As TA cells divide and differentiate, cells move up and out of the crypt and toward the apex of the villi or colonic lining, where, after 3–4 days, they undergo programmed cell death known as anoikis.

All that being said, cellular identity in the intestinal epithelium is highly plastic owing to broadly permissive chromatin signatures [24]. All of the partially differentiated and many of the fully differentiated cells types are capable of replenishing the Lgr5+ ISC pool in response to damage [25]. This is highly advantageous since an intestinal epithelium lacking active stems cells would prove quickly fatal. It was generally accepted that ISCs divide symmetrically, either proliferating to produce two stem cells or differentiating to produce two progenitor cells [26]. However, as newer computational models seem to favor more asymmetric division, this is a subject of continued debate; though both models agree that the stochastic division strategy favored in the intestine helps to protect the tissue from the accumulation of defects in the stem cell population, supporting the model of neutral drift [26,27].

While a multitude of growth factors and signaling cascades are involved in the proliferation and differentiation of stem cells into the various cell types, metabolism also plays a role in this process [28]. Like many other proliferating stem cells, it is thought that ISC rely on aerobic glycolysis to produce intermediates for the anabolic reactions necessary for cell division, whereas differentiated cells rely more on mitochondrial respiration [29,30]. Compared to other crypt base cells, Lgr5+ stem cells are particularly susceptible to electron transport chain inhibitors, which reduce oxidative phosphorylation and the ability to differentiate to form organoids [31]. Forcing flux toward or away from mitochondrial respiration directly influences cellular fate. The mitochondrial pyruvate carrier (MPC) is normally expressed at low levels in ISC. MPC deletion increases reliance on aerobic glycolysis resulting in increased stem cell proliferation and tumorigenicity in mice and flies, whereas overexpression increases mitochondrial respiration and results in a loss of ISC [32,33]. Other perturbations in pyruvate metabolism have been shown to skew differentiation toward the secretory lineage [34]. Interestingly, differentiation in skeletal myoblasts has also been linked to proper functioning of the electron transport chain, whose assembly is disrupted upon inhibition of mitochondrial fatty acid synthesis [35,36].

A dependence on metabolically active mitochondria for cell fate decisions has been identified in multiple stem cell types, including ISCs [37,38]. Mitochondrial dynamics appear to contribute to asymmetric stem cell division and are intimately linked with metabolic programming, cell cycle progression, and cell fate decisions in multiple cell types [39,40]. Mitochondrial fusion is often associated with increased oxidative phosphorylation and proliferation, while mitochondrial fission is associated with aerobic glycolysis and pluripotency. This does not mean that stem cells, like ISC, do not also utilize oxidative phosphorylation, but its use is likely tightly regulated and tied to cell fate commitment [41,42,43]. It is becoming clear that perturbed mitochondrial organization and dynamics also play a role in IBD [44,45,46]. The loss of the mitochondrial chaperone, HSP60, reduces mitochondrial function resulting in loss of proliferation and stemness in crypts and a concomitant increase in epithelial release of WNT10A and RSPO1 [38]. In worms, reduced expression of another chaperone, mitochondrial HSP70, results in activation of a mitochondrial-to-cytosolic stress response pathway which causes feedback inhibition of β-oxidation and a dramatic increase in lipid biosynthesis and storage [47]. Active mitochondria and damage response mechanisms are both required for proper stem cell function. ISC metabolism must balance a proliferating cell’s need for glycolytically derived anabolic substrates and mitochondrial metabolism to promote the differentiation that produces the full complement of cells needed for tissue homeostasis.

## 3. Lipid Metabolism in the Intestinal Epithelium

There is growing evidence that lipid metabolism plays an important role in the regulation of stem cells and TA cells in the small intestine [48,49,50,51]. It is believed that this regulation is due to both their function as an energy substrate and as a signaling molecule. Lipids, in the form of phospholipids and fatty acids, can be sensed via binding to nuclear hormone receptors (e.g., peroxisome proliferator-activated receptor [PPARs] and hepatocyte nuclear factor 4 [HFN4]), which serve as transcription factors and regulate the expression of many genes involved in lipid metabolism, inflammation, oxidative stress, and cellular differentiation [52]. Synthetic ligands (e.g., thiazolidinedione-type drugs) for these nuclear receptor proteins are commonly used to treat various metabolic diseases, including hyperlipidemia, diabetes, and obesity. Both lipid degradation and synthesis alter the pool size of other metabolites, like acetyl coenzyme A (acetyl-CoA), which serve as molecular rheostats that control metabolic flux [53]. Acetyl-CoA also serves as a precursor for post-translational histone modification, which can influence transcription of a variety of lipid and non-lipid related genes. Thus, lipids can impact transcription in their whole form as protein agonists or by feeding into metabolic pathways that impact deoxyribonucleic acid (DNA) accessibility via histone modifications. Additionally, lipid distribution in cellular membranes can affect the function of organelles. Next, we will discuss several recent examples of these mechanisms at work in the within the intestine.

Cpt1a catalyzes an early essential step in mitochondrial fatty acid oxidation (FAO), and is particularly important for long term homeostasis in the small intestine since its loss results in crypt apoptosis and reduced stem cell function [49]. Treatment of worms with the FAO inhibitor perhexiline results in fat accumulation, upregulation of the cytosolic heat shock response, and activation of the mitochondrial unfolded protein response, thus linking cellular stress and FAO inhibition [47]. Short-term fasting or treatment with a PPARδ agonist are both capable of enhancing stem and progenitor cell function under normal and aged conditions by upregulating FAO [49]. A high fat diet (HFD) also stimulates PPARδ and results in expansion of the proliferative compartment within the intestinal crypt and increases tumorigenesis [48]. In hematopoietic stem cells, PPARδ and FAO drive the asymmetric division essential for stem cell maintenance, but it is unclear if the same mechanism is at work in the intestine [39]. The PPARs work with PPARγ coactivator 1α (PGC-1α) to regulate insulin sensitivity and lipid handling by transcriptionally regulating mitochondrial biogenesis, fatty acid metabolism and reactive oxygen species scavenging gene programs [54]. Another transcription factor that regulates FAO predominantly in the progenitor population of the proximal small intestine is PRDM16, without which these cells apoptose and fail to create healthy differentiated tissue [50].

While usually thought of in context of the liver, the nuclear hormone receptor HNF4 is also highly expressed in the intestinal epithelium and directly activates genes that regulate FAO, and its absence causes a shift away from stem cell self-renewal toward rapid expansion of the progenitor compartment [51]. The negative effects associated with the loss of these transcription factors can be rescued by supplementing with acetate, which suggests that stem cell self-renewal and differentiation may be mediated by acetyl-CoA, the shared byproduct of both acetate metabolism and FAO [55]. Acetyl-CoA is a central metabolite also serving as a substrate in mitochondrial and cellular fatty acid biosynthesis, ketogenesis, the tricarboxylic acid (TCA) cycle, and as a co-factor for lysine acetyl transferases. Paracrine signaling within the intestinal crypt also helps to regulate stem cell function [15]. While short-term fasting induces FAO, the intestinal response to long-term calorie restriction causes changes in other related sensor metabolite pools [49]. Paneth cells sense calorie restriction and promote an increase the number of stem cells while also reducing the number of fully differentiated enterocytes in the intestine by increasing NAD+ production in ISC in an mTORC1- and SIRT1-dependent way [56,57]. Age-related loss of ISC function can be restored by replenishing NAD+ levels or by inducing FAO, hinting at overlap in these pathways [58]. Just as with increased FAO stimulation, there is a fine line between benefit and harm since excess NAD+ is also linked with increased tumorigenesis [59]. Their pro-proliferative effect also makes them possible targets for the treatment of intestinal cancers [60].

The connection between FAO and stem cell function in the colon is less well characterized, but a story about colonic metabolism is incomplete without describing the importance of SCFAs. SCFAs are present at millimolar concentrations in the intestinal lumen and are produced by microbial fermentation of indigestible fibers and some amino acids. Acetate, propionate, and butyrate are the three most abundant SCFAs produced in the gut and each play important roles in host homeostasis. Acetate and propionate are produced mostly by bacteria of the *Bacteroidetes* phylum, and butyrate is mostly produced by *Firmicutes* (*Roseburia* and *Clostridium*) [1]. Acetate is absorbed and used throughout the body as an energy source. Propionate is a weak histone deacetylase (HDAC) inhibitor, is able to enter metabolism via succinyl-CoA in the TCA cycle, and has been shown to play a role in hepatic/intestinal gluconeogenesis and satiety [61]. Of the three, butyrate has been investigated the most for its health benefits, which are largely attributable to its function as a strong HDAC inhibitor and as the primary energy source colonocytes [62].

FAO of butyrate to CO_2_, acetyl-CoA, and subsequently ketone bodies, provides 70% of their energy needs [63,64]. Experiments in germ-free (GF) mice have demonstrated that colonocytes induce autophagy to cope with the lack of microbially derived nutrition and signaling [65]. Colonocyte consumption of butyrate results in the formation of two gradients within the intestine: one decreasing along the tract from the proximal to the distal end, and the other decreasing from the lumen to the crypt base [66]. In fact, the structure of the crypt serves a protective role since high concentrations of butyrate cause HDAC mediated toxicity in highly proliferative cells like ISC and cancer cells [66,67]. Colonocytes utilize butyrate to fuel oxidative phosphorylation which helps maintain the anaerobic environment that supports the growth of most commensal bacteria [68]. Reliance on mitochondrial respiration increases the possibility of reactive oxygen species (ROS) production, which can alter the microbial diversity of the gut. This may partially explain the health benefits of dietary antioxidants [69,70]. In rats and humans, deficiency in FAO through inhibition or lack of SCFAs can induce colitis, a known risk factor for colorectal cancer [71]. Given the known benefits of butyrate for intestinal health, and the decreasing gradient within the colon, it is unsurprising that the incidence of colon cancers rises the closer you get to the rectum, where butyrate concentration is lowest. Colonocytes are also capable of metabolizing longer chain fatty acids and acylcarnitines that are present in bile and serum, which may act as a host-derived substitute when SCFAs are scarce [46].

We have already mentioned that mitochondria play an important role in cellular fate determination, and this can be partly attributed to their role in lipid metabolism. Mitochondria are the site of fatty acid β-oxidation, the location of mitochondrial fatty acid synthesis, the source of acetyl-CoA for cytosolic fatty acid and cholesterol synthesis, and the site of synthesis for various oxysterols, steroids, and bile acids [72,73]. In the intestinal lumen, dietary TGs are broken down and solubilized with bile salts to form micelles that dock with the apical side of the intestinal epithelium, thus delivering fatty acids to cells for mitochondrial β-oxidation [74]. While some of these fatty acids are catabolized, others reform as TG to be stored in intracellular lipid droplets or packaged into chylomicrons for transport through the blood [74]. Mitochondrial function is intimately linked to intracellular lipid composition and concentration, and may be disturbed when challenged by a high influx of dietary fat. The unique cristae structure of mitochondria depends on the asymmetric distribution of cardiolipin and phosphatidylethanolamine and phosphatidylcholine on opposing leaflets of the lipid bilayer [75]. The presence of these same phospholipids also influences the dynamics of fission and fusion of mitochondrial membranes, which have been linked to cellular fate in other contexts [76]. The lipid composition of mitochondrial membranes also stabilizes membrane proteins necessary for the electron transport chain and various other metabolic functions [76]. Mitochondrial membranes have lower concentrations of cholesterol than cellular or endoplasmic reticulum membranes; a higher-than-normal concentration within the mitochondrial membrane would likely alter mitochondrial function [72].

A cholesterol-rich diet also appears to increase proliferation in intestinal stem and progenitor cells, but in an FAO independent way [77]. In *Drosophila*, the amount of dietary cholesterol influences the development of secretory enteroendocrine cells in the fly midgut by modulating Notch and Notch ligand (Delta) levels, and kinetics [78]. A ketogenic diet is sometimes considered a healthy HFD, which aims to keep the body in a ketogenic state, typically seen during fasting, that favors the utilization of fats for energy instead of carbohydrates. The ketogenic HFD promotes ISC self-renewal and supports enterocyte differentiation via beta-hydroxybutyrate-mediated Notch signaling [79]. An unhealthy diet is among the biggest risk factors for the development of intestinal disorders like IBD and colorectal cancer and of bodily metabolic disorders. Mice fed a “total Western diet”, formulated based on National Health and Nutrition Examination Survey (NHANES) data to mimic the typical American diet in micro and macronutrient content, were at increased risk for developing both colitis and colorectal cancer [80]. Researchers have shown that colon cancer stem cell expansion and metastasis induced by a HFD is mediated by PPARδ effects on Nanog expression [81]. Diet-derived long chain unsaturated fatty acids appear to serve as PPARγ agonists, which regulate lipid metabolism in the intestine and bodily fat storage [82]. Under calorie restriction, intestinal PPARγ promotes sympathetic nervous system activity and mobilization of stored fat [83]. While in other tissues, the story about lipid metabolism might end here, in the gut we must consider the additional impact on the microbiome.

## 4. Diet Influences Microbial Community

As mentioned before, the gut microbiota is populated by bacteria from the *Firmicutes*, *Bacteroidetes*, *Actinobacteria*, *Proteobacteria*, *Verrucomicrobia* and *Fusobacteria* phyla, though certain regions of the gut favor certain species [2,17]. Together, *Firmicutes* and *Bacteroidetes* make up to 90% of the gut microbial population. A higher *Firmicutes* to *Bacteroidetes* ratio is commonly considered a biomarker for metabolic syndrome and obesity, but also increases with age [84,85]. Additionally, a *Bacteroidetes* population that favors *Prevotella* over *Bacteroides* is generally considered healthier than the opposite. Most of the pathogenic bacteria in the gut come from the *Proteobacteria* phyla, so it is also considered beneficial to maintain a low concentration of those inhabitants.

Taxonomic profiling of gut microbial communities is largely accomplished by sequencing microbial DNA from gastrointestinal samples. Fecal samples are the most accessible and utilized source of microbial DNA for gut microbiota studies, but they do not fully reflect the microbial diversity at different sites along the gastrointestinal tract [17]. While there are methods for sampling other sites, they are typically invasive and so are not usually appropriate for healthy individuals [86]. After DNA isolation, microbial amplicon sequencing, usually of the 16S rRNA gene, and/or whole metagenome shotgun sequencing, are used to determine which taxa are present and their relative abundances [87]. Each has their advantages and disadvantages in terms of resolution, cost, and technical difficulty. New direct hybridization methods may simplify the process [88]. Other technologies, like the BactoChip microarray and culturomics, allow for more granular investigation of diversity by allowing for species and strain level identification within a sample [89,90].

Microbes vary in their ability to metabolize different substances. Those that can rapidly grow on the available nutrients in an environment will dominate the microbial community, if only transiently. Just as diet, calorie restriction, and fasting can have an impact on intestinal cell biology, these also impact the gut microbiota [91]. Under nutrient rich conditions, microbes metabolize their preferred substrate, often via rapid fermentation and excretion, to replicate. These fermentation byproducts are metabolized by other bacteria who may then produce other metabolites that are utilized by yet other cells. This phenomenon is known as cross-feeding and happens between microbes within the gastrointestinal tract and between microbes and cells of the gastrointestinal tract.

Ultimately, nutrient availability in the gastrointestinal tract is decided by the host’s dietary choices. The standard Western diet is associated with a lower biodiversity than the gut microbiome of modern-day hunter gatherer societies, whose biomes fluctuate based on dietary changes resulting from seasonal food availability [92,93,94]. Even within industrialized societies there are some differences in the microbiomes between individuals consuming a vegan versus an omnivore diet [95]. However, these are blunted compared to traditional societies, possibly due to the loss of species diversity over generations of industrialized eating habits [10]. It has been suggested that there are a limited number of stable host–microbe symbiotic states, dubbed enterotypes, that are defined by their predominant species composition; *Bacteroides*, *Prevotella*, and other more disputed groups [96,97]. Long-term dietary patterns appear to be a major determinant of enterotypes, with high intake of protein and animal fat being characteristic of individuals who fall into the *Bacteroides* enterotype versus a high carbohydrate diet characterizing the *Prevotella* enterotype [98]. While moderate dietary changes can prompt rapid remodeling of the gut microbiota, these changes are not usually sufficient to cause enterotype switching. However, it has been observed as result of more extreme changes, like immigration to a more Western society, which results in an overall decline in diversity, a shift in dominance from *Prevotella* to *Bacteroides*, and an increase in obesity [99,100].

As already mentioned, variances in dietary patterns can influence the composition of the gut community. A HFD can alter the *Firmicutes* to *Bacteroidetes* ratio to mimic that seen in obesity even when there is no observable obesity phenotype [101]. While the modern Western diet, high in protein and fat, generally has a negative impact on the gut microbiota, there are modern diets, like the Mediterranean diet, characterized by a higher than typical consumption of cereals, fruit, vegetables, and legumes, which have a beneficial effect on the gut microbial profile [102]. Even intermittent deviations from a high-fat diet to a low calorie, synbiotic diet has been shown to have a beneficial effect on the diversity of the gut microbiota in mice [103]. Other modern diets, like the popular ketogenic diet, have their own specific effects on the composition of the gut microbiota [104]. So, while long-term dietary patterns define the dominant gut composition, even small dietary changes can influence the gut microbiota. Forming new dietary patterns may enable a shift in the balance toward more beneficial microbes with the associated health benefits thereof, though probiotic supplementation may be necessary to repopulate extinct species.

A HFD can cause overflow of dietary fats to the distal portion of the intestine, causing a shift in the balance away from glycan degraders (*Clostridium*, *Eubacterium/Roseburia*) toward fatty acid degraders (*Alistipes*, *Bilophila*), which lowers the production of beneficial SCFA and antioxidants in the gut [105,106]. The resulting altered luminal environment can support the growth of pathogenic bacteria that produce proinflammatory endotoxins, like lipopolysaccharides. While all HFDs appear to influence microbial diversity to some extent, the specific effect on a microbial community can depend on the proportion of saturated versus unsaturated fats in the diet, their chain length, their degree of unsaturation, and the location of double-bonded carbons within the chain [106,107,108,109,110]. The increased *Firmicutes* to *Bacteroidetes* ratio typical of obesity is more associated with consumption of saturated versus unsaturated fats [106]. Monounsaturated fatty acids, like oleic acid found in olive oil, are associated with increased biodiversity in the gut and a more favorable *Firmicutes* to *Bacteroidetes* ratio [108]. Polyunsaturated fats (PUFAs) have a growth inhibitory effect on many bacterial species [111]. However, some PUFAs are partially metabolized in the distal intestine by *Bifidobacteria* and *Lactobacilli*, which facilitates their ability to outcompete pathogenic *Enterobacteria* [112,113]. The optimal dietary ratio of omega-6 to omega-3 PUFAs is 1–4:1; however, the modern Western diet often contains a ratio closer to 20:1, which increases the risk for obesity, cardiovascular disease, and cancer [113,114]. While many studies have shown that PUFAs are largely beneficial to human health and have a positive impact on the gut microbiome, other research suggests that omega-6s increase gut dysbiosis and colitis risk [108]. Results are not always consistent across different studies due to variations in study design, natural variations in endogenous microbiomes of individuals, and unanticipated growth effects in response to different lipids, but there is evidence that a HFD alters the normal balance within the gut. The question then becomes, how do microbes interact with the lipids in their environment?

## 5. Microbes Metabolize Dietary Lipids

We have also seen that a HFD can cause changes in the abundance of different microbial species in the gut that are associated with disease. While short-term HFD-induced changes can be reversed by changing dietary habits, a long-term commitment to a HFD establishes a permanent shift in microbial diversity due to chronic underfeeding of some species and overfeeding of others. The availability of the lipids in the gut varies depending on location, as does microbial abundance and composition. The proximal small intestine is the most lipid-rich environment in the bowel and is largely populated by a dynamic cast of *Firmicutes* and *Proteobacteria*, changing in response to daily dietary nutrient intake [115]. In the colon, fiber derived SCFAs from microbes are preferentially taken up by colonocytes and protect against HFD-induced obesity colonocytes [1,46].

Gut microbial species like *Lactobacillus plantarum* and *Bifidobacterium breve* can conjugate the PUFAs, linolenic and γ-linolenic acid, via a series of hydroxy, oxo and partially saturated trans-fatty acid intermediates [114]. The ileum and colon of GF mice, which lack the enzymes responsible to catalyze the formation of conjugated linoleic acid, contain higher concentrations of PUFAs and PUFA-containing phospholipids [116]. These oxo and hydroxy-fatty acids have anti-obesity and anti-inflammatory effects, as well as other effects that benefit the host [116]. Inspired by these microbial products, researchers have made synthetic “enone” fatty acids, which ameliorate the effects of inflammation in adipose tissue [117].

In the distal small intestine and colon, where microbial populations are higher, microbes lower luminal concentrations of cholesterol and elevate levels of phosphatidylcholine [116]. Several *Lactobacillus* species, like those found in fermented milk and traditional Chinese pickles, protect against diet-induced obesity by metabolizing luminal cholesterol, thus reducing the cholesterol available for absorption by intestinal enterocytes [118,119]. Other bacterial strains have been identified among the genus *Eubacterium* who convert cholesterol to coprostanol, which is not absorbed but excreted via feces [120]. A recent paper identified *ismA* as the microbial gene that encodes the cholesterol dehydrogenase responsible for this conversion. This gene’s presence in the microbiome is associated with lower serum cholesterol levels in humans and reduced risk of cardiovascular disease [121]. An in vitro study in an enteroendocrine cell line demonstrated that the fatty acid receptor GPR40 stimulates potent cholecystokinin (CCK)-release in response to the presence of specific oxo-fatty acids produced by lactic acid bacteria [122]. CCK and secretin are two peptide hormones that regulate the emulsification and digestion of fat through the release of pancreatic enzymes and control over gastric emptying. A study in GF mice suggests that while microbes are not required to stimulate the release of bile acids upon treatment with corn oil, they are required for pancreatic lipase release [115].

Bile acids, derived from cholesterol, are an important class of steroids that play a role in lipid metabolism in the gut. Bile acids can influence the composition of the gut microbiota of the intestine, and this composition influences the species of secondary bile acids available for host metabolism [123]. While most primary bile acids are reabsorbed in the distal small intestine, a small amount (5–10%) makes it to the colon, though this percentage can increase under a HFD [62]. Secondary bile acids act as important signaling molecules in the gut and throughout the body [120]. Reports vary as to whether these metabolites are more detrimental or beneficial. Lithocholic acid and deoxycholic acid, two secondary bile acids, are both associated with colon cancer risk, but also appear to play an important role in intestinal wound healing [120,124]. Crypt regeneration occurs when deoxycholate binds to the farnesoid X receptor (FXR), master regulator of bile acid homeostasis, and subsequently suppresses prostaglandin E2 synthesis [124]. Deoxycholic acid binding to FXR has a net pro-proliferative effect, which may help explain the correlation between secondary bile acids and colon cancer progression [125].

Sphingolipids also play a role in signaling, modulating inflammation, and immunity, and are important for cellular membrane structure. Several bacterial species from the *Bacteroidetes* phylum can synthesize sphingolipids starting from serine, alanine or glycine, and an acyl-CoA thioester. Microbially-produced sphingolipids help condition the infant immune system [126]. Host de novo production of sphingolipids is inversely proportional to microbial sphingolipid synthesis, suggesting that the host can sense sphingolipid levels and adjust production accordingly [127,128]. This is just one example of host–microbial crosstalk; next, we will explore more.

## 6. Microbial Impact on Host Lipid Metabolism

While some studies have focused on the way specific microbes interact with different lipids, others have focused on how microbial species influence host metabolism more globally. Although they constitute a relatively minor proportion of the microbiota, increased concentrations of *Akkermansia muciniphila* are correlated with healthier metabolic status and are protective against diet-induced obesity, suggesting that abundance is not the only metric worth consideration [18,19]. Probiotic treatment results in a small but consistent reduction in body weight, body mass index (BMI), and fat percentage in overweight and obese individuals compared to placebo controls [129].

Enterocytes can resynthesize TGs, which are either stored in lipid droplets or repackaged into chylomicrons and secreted into the lymphatic system [74]. Certain microbial metabolites have been shown to modulate this process by inhibiting chylomicron secretion via two different mechanisms. Lactate produced by *L. paracasei* promotes fatty acid synthesis and lipid storage, whereas acetate produced by *E. coli* promotes FAO [130]. Diglyceride acyltransferase (Dgat) 1 and Dgat2 are both capable of catalyzing the final committed step of TG synthesis, but their function in the body is non-equivalent, where increased Dgat2 is associated with higher rates of TG secretion [74]. A HFD alters the microbial profile and increases Dgat2 expression in specific pathogen free mice, but not in GF mice, suggesting that the Dgat2 response to a HFD is microbially mediated [115]. Together, these data help explain why GF mice have impaired lipid absorption and lower levels of circulating TG compared to specific pathogen-free mice [115]. The dysbiosis and altered lipid profiles resulting from a HFD are also associated with decreased intestinal motility resulting from cell death of myenteric neurons that express Toll-like receptor (TLR) 4 in the proximal colon [131].

Given the microbiota’s ability to influence TG and chylomicron secretion from enterocytes, it is not surprising that altered microbial composition influences circulating lipid levels in the host; in fact, computational modeling has been used to estimate that, independent of other factors, the microbiota is able to explain 4.5% of the variance in body mass index, 6% in TGs, and 4% in high-density lipoprotein [132]. A later study showed an obesity-specific association between the microbiota and lipid composition in very low-density, low-density, and intermediate-density lipoproteins [133].

The gut microbiota also effects more global lipid storage in the host. Researchers have recently identified δ-valerobetaine as a diet-dependent obesogen that is produced by a wide variety of intestinal microbes [134]. GF mice gain less weight on a normal chow diet than conventionalized mice despite increased consumption, suggesting that the gut microbiota is an important regulator of body fat storage [135]. There is a lot of variability in the data published on the effect of diet-induced obesity on GF mice, especially when it comes to a HFD. These variations may arise due to the composition of the fats provided in the diet. While GF mice fed a high-fat, lard-based diet appear resistant to diet-induced obesity, this effect was not seen when the HFD is palm oil-based, which the researchers hypothesized may be because the cholesterol present in the lard diet inhibits cholesterol biosynthesis [136]. In conventional mice, a lard-based diet is associated with increased body weight and white adipose tissue inflammation via TLR signaling [137]. When GF mice are inoculated with a microbial strain isolated from the gut of a morbidly obese human and then fed a HFD, they develop obesity, insulin resistance, and high levels of systemic inflammation [138]. Gene-ontology enrichment analysis of the differentially expressed genes seen in these mice compared to other experimental conditions identify major changes in inflammatory response and lipid, lipoprotein, and sterol metabolism [138]. As might be expected, the ability of the microbiota to influence fat storage in GF mice is dependent on the composition of the microbial community introduced, as the accumulation of fat mass was higher in mice inoculated with microbiota isolated from obese donors than from lean donors [139]. A human twin study found that visceral fat mass accumulation is linked to both gut microbiota composition and diet, but bacteria is associated with fat mass even independent of diet, a result that reiterates those from studies in GF mice [140].

The ability of the microbiome to impact fat storage was suggested by clinical association studies which report a positive correlation between the accumulation of microbially derived δ-valerobetaine and a high BMI [134]. Given this, it is not surprising that the gut microbiome has been implicated in diseases like atherosclerosis and non-alcoholic fatty liver diseases, whose pathologies are driven by circulating lipids and their storage [141,142]. Additionally, the gut microbiota also influences cardiovascular risk via the production of metabolites like trimethylamine-N-oxide (TMAO), secondary bile acids, and short-chain fatty acids [143]. Gut microbes can produce the metabolite trimethylamine (TMA) from precursors like phosphatidylcholine, L-carnitine, choline and betaine, which can be further metabolized by hepatic flavin monooxygenases to TMAO, a known driver of cardiovascular disease pathogenesis [143]. A recent paper linked the rise in TMA producing bacteria to the rise in luminal oxygen resulting from impaired mitochondrial bioenergetics following a HFD [144].

There is complex crosstalk between the host, the microbiome, and the diet. Certain disease states are associated with a specific bacterial signature. Individuals with hypercholesterolemia, a major risk factor in cardiovascular disease, have bacterial fecal profiles characterized by lower abundance of *Anaeroplasma* and *Haemophilus* and higher abundance of *Odoribacter* relative to individuals with normal cholesterol levels [145]. Transplantation of the microbiota from humans with hypercholesterolemia to recipient mice induced high plasma cholesterol levels in association with a low hepatic cholesterol synthesis and high intestinal absorption [146]. Host factors like diet, age, and hormonal changes associated with pregnancy and menopause can influence the gut microbiome and lipid-associated disease risk, making it difficult to unravel the driving factors [141,147]. While many of these factors are fixed, the microbiome can be modulated by dietary choices, and offers an attractive avenue for manipulating disease risk.

The microbiome can impact host lipid metabolism through manipulation of the same nuclear hormone receptors and upstream regulatory factors, like SIRT1 and PGC1α, that we already discussed [148,149]. As mentioned before, the obligate anaerobes of the gut and the host work together to maintain an oxygen-depleted luminal environment that prevents the expansion of potentially pathogenic facultative anaerobes [150]. This is accomplished, in part, because microbial metabolites induce PPARγ signaling, which promotes FAO and subsequent oxygen consumption by the electron transport chain [150]. Microbially-produced butyrate, conjugated linoleic acid, and intermediate fatty acids can influence the expression of different PPARs and impact barrier integrity and inflammatory conditions like colitis [150,151,152,153]. For instance, *Lactobacillus plantarum* protects against metabolic disorders by upregulating SIRT1, PPARα, and PGC-1α in the liver and adipose tissues of mice fed a HFD [148]. On the other hand, the loss of beneficial gut microbes and their metabolites negatively impact host lipid metabolism in the gut and in peripheral tissues. Salmonella-induced gastroenteritis results in dysbiosis and reprograms host metabolism, causing a shift away from butyrate oxidation toward pyruvate fermentation and lactate excretion, which further supports pathogenic growth [154]. A HFD induces changes in the gut microbiota, causing increased expression and oscillation of hepatic PPARγ and its downstream target genes, resulting in increased epididymal fat accumulation and hepatosteatosis in mice [155].

The nuclear receptor/transcription factor HNF4α is another identified player in lipid metabolism in the intestine. HNF4α has binding sites in the promoter region of multiple genes involved in glucose and lipid metabolism, and has also been implicated in the regulation of fatty acid uptake in enterocytes due to indirect modulation of fatty acid transport protein (FATP) 4 expression, an acyl-CoA synthetase with an affinity for long chain fatty acids (LCFA), and very long chain fatty acids (VLCFA) [156]. Interestingly, HNF4α has also been implicated in the transcriptional induction of other genes that synthesize VLCFAs and VLCFA-derived hydrocarbons in *Drosophila melanogaster* [157]. HNF4α and intestine-specific HNF4γ are mostly redundant and activate genes that regulate FAO; the double knockout results in loss of stem cells and differentiated enterocytes in the intestinal epithelia, again signifying the important role for FAO in tissue homeostasis [51]. The gut microbiota can regulate lipid metabolism by suppressing sequence-specific binding of the transcription factor HNF4α to cis-regulatory DNA, possibly through loss of poised (H3K4me1) and activated (H3K27ac) enhancers [149]. HNF4α activity can be also be activated by dietary LCFA and VLCFAs. Microbiota-dependent and independent suppression of HNF4α activity likely plays a role in the altered serum lipid profiles, absorption, and transport of lipids and cholesterol common in individuals with IBD, type 2 diabetes, and metabolic syndrome [149].

Long-term IBDs like ulcerative colitis and Crohn’s disease are risk factors for the development of colorectal cancer [158]. Throughout this paper, we have linked a HFD, increased FAO, and a dysbiotic microbiota to susceptibility of inflammatory bowel disease and colorectal cancer. Interestingly, while fat appears to be causative of disease in many cases, in Crohn’s disease, the body also appears to utilize “creeping fat” to protect itself from systemic distribution of pathogenic bacteria. It has been hypothesized that this fat acts as a physical barrier to impede bacterial transit across the weakened epithelial barrier in Crohn’s disease patients [159]. Indeed, others have also observed bacterial translocation to the creeping fat and mesenteric adipose tissue in Crohn’s disease patients, where it serves as a bacterial reservoir and correlates with disease status [160].

## 7. Microbiota, Lipid Metabolism, and Circadian Rhythms

Another way diet and gut microbes manipulate human health is through their interactions with normal circadian rhythms. In healthy humans, free fatty acids, TGs, and glucose all exhibit diurnal variations [161]. Circadian rhythms are entrained on the daily cycle of light caused by day and night, but food uptake in the gut is also a synchronizing factor for the circadian clock. The active period of the day is characterized by gathering and eating food, and the inactive period is marked by fasting and rest. Both a HFD and the ketogenic diet are known to have a dramatic effect on clock regulation in the body due to oscillations in PPARs and their target genes [162,163]. Energy-sensing transcription factors like PGC1α help reinforce circadian rhythms by stimulating the expression of the important clock gene, brain and muscle ARNT-like 1 (BMAL1) [161]. BMAL1 works with the Circadian Locomotor Output Cycles Kaput (CLOCK) protein to regulate the expression of many genes involved in lipid metabolism, including mitochondrial FAO and respiratory metabolism [164,165]. The now familiar HNF4α plays a different role in circadian oscillations in the intestine and liver, where it acts as transrepressor of the CLOCK:BMAL1 dimer [166]. Human biology is adapted for diurnal behavior, meaning our active periods are during the day. Different aspects of modern life (e.g., shift work, jet lag) can detrimentally impact the normal circadian cycle. Disruption of circadian rhythms increases the risk of developing many of the diseases we have already touched upon in this review, such as obesity, diabetes, neurodegeneration, and cardiovascular disease [166]. Recent studies have demonstrated that compared to day shift workers, night shift workers are more prone to obesity, impaired lipid metabolism, and gastrointestinal disorders, all of which we know are also linked to diet and the microbiome [167,168]. So far it is unclear whether shift work causes changes in the microbiome in humans, but there is evidence from mice that circadian disruption can cause dysbiosis [168,169,170].

The microbiota is impacted by and has an impact on host circadian cycles. In mice, it has been demonstrated that the microbiota exhibits cyclic variations in composition, function, and localization [171,172]. A HFD disturbs the normal diurnal patterns of microbial community structure and resulting function, which in turn may disrupt normal host circadian signaling, thus impacting host lipid metabolism and diet-induced obesity [171]. The effects of a HFD on clock reprogramming are largely attributed to de novo oscillations in PPARγ and its target genes [162]. Fecal transplant studies have since revealed that these HFD-dependent PPARγ-driven changes are mediated by changes in the gut microbial community [155]. Research suggests that one way the microbiota promotes adipose storage is by inducing NFIL3 expression in the small intestinal epithelium by repressing the circadian clock factor REV-ERBα [173]. Interestingly, it appears that an immune response to motile Gram-negative bacteria like *Salmonella typhimurium* and *Escherichia coli* stimulate this signaling cascade, providing another reason to consider these organisms among the “bad” gut bacteria [173]. This same group of researchers later demonstrated that the microbiota induces the cyclic expression of HDAC3 in the small intestine via the TLR adaptor protein MyD88, resulting in the cyclic acetylation and activation of many genes involved in nutrient transport and lipid metabolism [174]. The microbiota is also thought to regulate host metabolism in the intestine and liver via oscillations in microbially produced and modulated metabolites, like SCFAs and bile acids, which influence host epigenetics and transcription [175,176,177,178,179]. In addition, the microbial metabolite butyrate has a purported sleep-promoting effect which may also help reinforce proper circadian cycling [175].

## 8. Summary and Future Directions

Herein, we have reviewed how dietary nutrients affect host and gut microbiota both individually and sequentially. Diet and microbes are both independent risk factors for disease, but diet can also alter the composition of the gut microbiota. A HFD increases proliferation in the intestinal crypt, susceptibility to tumorigenesis, and risk of metabolic diseases, while also shifting the composition of a gut toward fatty acid degraders and away from glycan degraders. This results in an increased *Firmicutes* to *Bacteroidetes* ratio, independent of an observable obesity phenotype [101]. Over time, microbes that are not fed die off and are not passed on to future generations, resulting in loss of species diversity over time [10]. It is possible that even a “healthy” diet may fall short of its potential benefits if the host lacks the cohort of microbes to digest and cross-feed the host’s cells. It therefore becomes important to know not only which foods are beneficial to human health, but which probiotics may be necessary to make the most of that food. Fats are necessary nutrients, but what type and how much is still being investigated. Normal circadian biology depends on periods of feeding marked by reliance on carbohydrates and periods of fasting marked by reliance on stored lipids. Robust, healthy mitochondria are required for the metabolic flexibility that allows for easy switching between fuel sources [176]. The intestinal epithelium itself relies on mitochondrial FAO for stem cell function and tissue homeostasis. Microbes within the gut modulate the host’s response to dietary lipids by modifying these lipids and/or by guiding their use.

We are still in the early days of understanding the significance of changes in gut microbial ecology and whether certain populations of microbes exert more influence than others on the host under different dietary conditions. A number of whole-organism metabolomics studies have revealed preferential absorption and extraction of different nutrients by different organs [177,178,179,180], but these studies fail to capture the full extent of intestinal nutrient utilization since more of the energy supplied to this organ comes from the lumen than from the vascular system [14]. This means that the contribution of new dietary nutrients and microbial metabolites is missed. Future studies of the intestinal tract should therefore try to account for the microbial factor. Our understanding of lipid metabolism requires clear study designs when investigating the effects dietary lipids. Just as the human body does not respond to saturated and unsaturated fats the same way, neither do the microbes within the gut. The composition of a HFD may matter as much or more than its concentration. Ultimately, researchers are seeking to define which microbial species constitute a healthy microbiome, how those microbes are beneficial, and how to cultivate those microbes within the gut for disease prevention and even disease reversal.
